# Effectiveness and Safety of Nadroparin Therapy in Preterm and Term Neonates with Venous Thromboembolism

**DOI:** 10.3390/jcm10071483

**Published:** 2021-04-02

**Authors:** Jeanine Sol, Marit Boerma, Irene Klaassen, Sinno Simons, Bregje Witjes, Enno Wildschut, Irwin Reiss, Cornelia Heleen van Ommen

**Affiliations:** 1 Department of Pediatrics, Reinier de Graaf Gasthuis, 2625 AD Delft, The Netherlands; 2 Neonatal Intensive Care Unit, Erasmus MC Sophia Children’s Hospital, 3015 CN Rotterdam, The Netherlands; s.simons@erasmusmc.nl (S.S.); i.reiss@erasmusmc.nl (I.R.); 3 Department of Pediatric Hematology, Erasmus MC Sophia Children’s Hospital, 3015 CN Rotterdam, The Netherlands; marit_boerma@hotmail.com (M.B.); c.vanommen@erasmusmc.nl (C.H.v.O.); 4 Department of Pediatric Hematology, Amsterdam University Medical Center, 1105 AZ Amsterdam, The Netherlands; i.l.klaassen@amc.uva.nl; 5 Department of Clinical Pharmacy, Erasmus Medical Center, 3015 GD Rotterdam, The Netherlands; b.witjes@erasmusmc.nl; 6 Pediatric Intensive Care Unit, Erasmus MC Sophia Children’s Hospital, 3015 CN Rotterdam, The Netherlands; e.wildschut@erasmusmc.nl

**Keywords:** neonate, thrombosis, nadroparin, therapeutic target range, effectiveness and safety

## Abstract

Introduction: Optimal neonatal nadroparin dosages to treat venous thromboembolism (VTE) are unknown. Objective: To evaluate therapeutic nadroparin dosages to reach therapeutic target ranges (TTR: 0.5–1.0 International Unit (IU)/mL) and the effectiveness and safety of nadroparin in neonatal VTE. Methods: Retrospective study including neonates with VTE on nadroparin in a tertiary center between 2007 and 2018. Two groups were distinguished: neonates before (group 1) and after (group 2) switch to higher starting dosages in 2014. Results: Sixty-one neonates (44 preterm, 17 term) with 64 VTEs were included. TTR was reached in 32/64 (50%) VTEs (group 1: 35.7%; group 2: 61.1%). Median nadroparin dosage to reach TTR was 197 (97.9–330.3) IU/kg/12 h. No therapy-related deaths occurred. Recurrent VTE developed in 6 (9.8%) neonates. Complete clot resolution was observed in 31/41 (75.6%) VTEs. TTR was reached in 58.1% VTEs with complete clot resolution. No major bleeding occurred. Non-major clinically relevant bleedings occurred in 3/64 (4.7%) VTEs, consisting of large hematomas due to the use of subcutaneous catheters. Conclusions: High nadroparin dosages are needed to reach TTR in neonates, which seem to be safe. Clot resolution may occur without reaching TTR. Subcutaneous catheters may cause important bleeding complications.

## 1. Introduction

The incidence of venous thromboembolic events (VTEs) in preterm and term neonates is rising [1]. This is caused by an increased awareness, improved diagnostic methods, increased survival of critically ill preterm, and term neonates, and advances in care, including the use of central venous catheters [1,2]. In neonates, more than 90% of thrombi are catheter-related [3,4].

Low-molecular-weight-heparin (LMWH) has become the anticoagulant of choice in neonatal VTE [2,5,6,7]. It potentially offers advantages over unfractionated heparin (UFH), such as a reduced risk of bleeding, subcutaneous administration, increased bioavailability, more predictable dosage response and less frequent monitoring. Lack of prospective clinical trials of LMWH in neonates has led to extrapolation of results of adult management studies to children. However, extrapolation of adult results to neonates is difficult due to differences between neonatal and adult hemostasis. This may be dangerous because of the presence of severe underlying medical conditions, including a high risk for intraventricular hemorrhages, that increases the risk of bleeding complications in these vulnerable infants [8].

Controversies exist over the current optimal dosages of LMWH in neonates. Compared to adults, children require higher dosages of all LWMHs with decreasing age to reach the therapeutic target ranges (TTRs) [9,10]. For example, for enoxaparin, which is the most studied LMWH in children, a recent review reported a mean dosage of 2.1 mg/kg/12 h in premature neonates, and 1.0 mg/kg/12 h for children from 6 years onwards to reach TTR [11]. Data about dosages to reach TTR as well as efficacy and safety of nadroparin in neonates are scarce. Only one study prospectively studied 76 patients from 0 to 18 years old, with a starting nadroparin dosage of 85.5 International Unit (IU)/kg/12 h, including 12 neonates [12]. Those neonates eventually needed a mean dosage of 224 IU/kg/12 h to reach TTR of 0.5 to 1.0 IU/mL. Furthermore, they required significantly more time to reach TTR than older children. In the national Dutch pediatric VTE guideline of 2014, the starting dosage of nadroparin was, therefore, increased in neonates. In this retrospective cohort study, we aimed to evaluate the effectiveness and safety of nadroparin in preterm and term neonates.

## 2. Materials and Methods

### 2.1. Study Design

This is a single center, retrospective observational cohort study, which was performed in the Erasmus MC Sophia Children’s Hospital in Rotterdam, the Netherlands. The local Medical Ethics Review Committee confirmed that official approval of this study was not required as the Medical Research Involving Human Subjects Act did not apply to this study.

### 2.2. Patient Population

In our institution, nadroparin is prescribed for initial treatment of neonates with VTE, including cerebral sinovenous thrombosis.

Between 1 January 2007, and 31 December 2018, all preterm and term neonates (<2 months corrected age) with suspicion and/or diagnosis of VTE, who received nadroparin (Fraxiparine^®®^) were included in this study. Patients who did not undergo measurements of anti-Xa activity levels were excluded, as well as patients with missing data on nadroparin dosages.

The included patients were divided into two groups depending on the type of protocol used (before or after protocol switch in 2014). Group 1 was defined as all included patients treated with nadroparin between 2007 and 31 December 2013. Group 2 was defined as all included patients treated with nadroparin between 2014 and 2019.

### 2.3. Data Collection

Patients with neonatal VTE were selected from the thrombosis database of the pediatric hematology department and by a search of the hospital admission database using the following filters: “nadroparin”, “anti-Xa-level”, “thrombosis”, and “neonates”.

The following data were retrospectively obtained from medical electronic records and computerized patient data management systems: gestational age, sex, birth weight, very low birth weight (VLBW) neonates (<1500 g), extremely low birth weight (ELBW) (<1000 g), age at start nadroparin therapy, weight at start nadroparin therapy, location of VTE, associated clinical risk factors (the presence of a central venous catheter, suspicion of sepsis, congenital heart disease), initial nadroparin dosage (IU/kg/12 h), anti-Xa levels (IU/mL) and corresponding nadroparin dosages (IU/kg/12 h), date of anti-Xa levels, time to achieve TTR (days), number of dosage adjustments, recurrent thrombosis, clot resolution, complications of nadroparin therapy (bleeding and allergic reaction), complications of subcutaneous catheters, complications of neonatal VTE (pulmonary embolism, stroke, and death due to VTE).

### 2.4. Anticoagulation Protocol

Until January 2014, most neonatal thrombi were treated with nadroparin. Relative contraindications included invasive surgical procedures and intracranial bleedings in the preceding 10 days, and active bleeding. In 2014 the national protocol for treatment of catheter-related neonatal VTE was introduced [13]. In this protocol, a wait and see policy is applicable for nonocclusive thrombi in the veins and small thrombi in the right atrium (less than half of the atrium). If extension occurs on ultrasonography, prompt start of nadroparin should occur. Occlusive thrombi in the veins and large atrial thrombi are treated with nadroparin immediately. Re-evaluation by ultrasonography is performed after 6 weeks of anticoagulation. The maximum duration of nadroparin therapy in neonatal VTE was three months. If at an earlier stage ultrasonography showed that thrombosis had resolved, nadroparin therapy could be stopped.

From 2007 to 2014, the advised starting dosage of nadroparin for preterm and term neonates treated for VTE in the local written thrombosis policy was 85 IU/kg/12 h, which was extrapolated from adult starting dosage. In 2014, this dosage was increased to 150–180 IU/kg/12 h. Standard nadroparin dosages were diluted with 0.9% sodium chloride solutions to make specific dosages of nadroparin for every child. A volume of 0.2 mL was the minimum that could be administered via a subcutaneous catheter. After start and dose adjustment of nadroparin therapy, anti-Xa levels were usually measured after the second or third administered dosage. Blood samples were taken four hours after nadroparin administration.

### 2.5. Coagulation Assays

The blood was collected by venipuncture in 3.2% trisodium citrate tubes and directly analyzed in the hematology laboratory of Erasmus MC Rotterdam. Firstly, the citrate tubes were centrifuged for a quarter of an hour at 1700 gravitational-force. The anticoagulation to blood ratio was 0.1/0.9 (*v*/*v*). Anti-Xa levels were analyzed in all blood samples by using the Sysmex CS5100 system (Siemens Healthineers, Erlangen, Germany). Antithrombin and chromogene substrate (S2732) were added to the trisodium citrate tubes, so inactive complexes between anti-Xa factor and antithrombin were formed. The residual fragments of anti-Xa were measured in all blood samples and were used as the represented anti-Xa levels in the body.

### 2.6. Outcome Parameters

#### 2.6.1. Therapeutic Target Range

Endpoints of nadroparin treatment included the median nadroparin dosage (IU/kg/12 h) to achieve therapeutic target anti-Xa activity levels between 0.5 and 1.0 IU/mL [5]. Other endpoints were time and number of dosage adjustments needed to reach TTR. Time to reach TTR was measured in days with a cutoff point at 21 days after start nadroparin therapy.

#### 2.6.2. Effectiveness and Safety

Effectiveness primary outcomes included death due to VTE and recurrent VTE. Recurrent VTE was defined as radiologic confirmation of extension of the current thrombosis, or development of a new symptomatic VTE during nadroparin therapy. Effectiveness secondary outcome included clot resolution, defined as no detection of residual thrombosis by radiological follow-up. Neonatal VTEs were excluded for this outcome parameter if prior to or after early discontinuation of nadroparin therapy, another anticoagulant or thrombolytic agent was given.

Safety outcomes included the incidence of major bleeding, and clinically relevant non-major bleeding (CRNMB) and minor bleeding. Definitions were according to criteria of the Perinatal and Pediatric Haemostatis Subcommittee of the Scientific and Standardization Committee (SSC) of the International Society on Thrombosis and Haemostatis (ISTH) as reported by Mitchell et al. [14] Major bleeding was defined as (1) fatal bleeding, (2) clinically overt bleeding associated with a decrease in hemoglobin of at least 20 g/L (i.e., 2 g/dL or 1.24 mmol/L) in a 24-h period, (3) bleeding that is retroperitoneal or pulmonary, or (4) bleeding that requires surgical intervention in an operating room. Intracranial bleeding is categorized major bleeding as defined by Curley et al. in the Planet-2 study [15]. All intracranial bleedings which were not defined as major bleeding will be categorized as non-major intracranial bleeding. CRNMB was defined as a composite of (1) overt bleeding for which a blood product is administered and not directly attributable to the patient’s underlying medical condition and (2) bleeding that requires medical or surgical intervention to restore hemostasis, other than in an operating room [14]. Minor bleeding was defined as any overt or macroscopic evidence of bleeding that does not fulfil the above criteria for either major bleeding or CRNMB [14]. Additional significant complications such as allergic skin reaction, and subcutaneous catheter problems were notified when presented during therapy.

### 2.7. Data Statistical Analysis

Baseline characteristics, pharmacokinetic and pharmacodynamic data were analyzed by descriptive statistics. Differences between group 1 and group 2 with regard to patient or VTE episode characteristics were analyzed by Mann–Whitney U tests for continuous variables and by Chi-Squared tests for categorical data. Pharmacokinetic data were presented as median and ranges (minimum and maximum). Subgroup analyses of pharmacokinetic outcomes were performed by different statistical tests, including Chi-Squared tests and Mann–Whitney *U* tests. Pharmacodynamic data outcomes were presented as frequencies and their percentages. A significance level of *p* < 0.05 was used in all tests. Data were managed and analyzed using IBM SPSS Statistics 27.0 (IBM, Armonk, NY, USA).

## 3. Results

### 3.1. Patient Characteristics

In this retrospective study, 61 neonates with 64 VTEs were enrolled. Twenty-seven (44.3%) neonates were treated before 2014 and 34 (55.7%) neonates after 2014. Three neonates were included twice as anticoagulation therapy was switched from LMWH to UFH for a short period of time due to the occurrence of recurrent thrombosis (*n* = 2) and subcutaneous catheter complication (*n* = 1). Baseline characteristics of the included patients are presented in Table 1. Ten neonates had cerebral sinovenous thrombosis. Six of these infants were described in the study of Raets et al., a prospective observational study with cranial ultrasonography in preterm infants less than 29 weeks of gestational age [16]. In total, 44 preterm neonates (gestational age < 37 weeks) and 17 term neonates (gestational age ≥ 37 weeks) were included in this study. Almost 60% were VLBW (*n* = 11) and ELBW (*n* = 24) neonates. Median postnatal age at the start of nadroparin therapy was 16 days, with a wide range from 1 to 124 days. Weight at the start of nadroparin therapy was below 1500 g (*n* = 15) and below 1000 g (*n* = 14) in 45% of the neonatal VTEs. In group 1, significantly more preterm and, as a consequence, more VLBW and ELBW neonates were included. All neonates had a normal renal function at start nadroparin therapy and platelets above 30 × 10^9^/L.

### 3.2. Therapeutic Target Range

TTR data are presented in Table 2. TTR was reached directly after the initial dosage in 11 neonatal VTEs. Finally, after dosage adjustments, therapeutic levels of nadroparin were reached in 32 of 64 (50%) neonatal VTEs. The median nadroparin dosage to reach TTR was 197.0 (97.9–330.3) IU/kg/12 h. Time to reach TTR varied from 1 to 21 days, with a median of 3.5 days. TTR was significantly reached more often (*p* = 0.044) in neonates (*n* = 22) with a weight above 1500 g at the start of nadroparin therapy. Median nadroparin dosage to reach TTR was not significantly higher, 198.8 (111.1–330.3) IU/kg/12 h in this group versus 180.6 (97.9–273.5) IU/kg/12 h in neonates below 1500 g and 1000 g. The median dose to achieve TTR was 181.1 (97.9–220.6) IU/kg/12 h for the neonates below 1500 g and 180.0 (124.5–274) IU/kg/12 h for the neonates below 1000 g, respectively.

Due to various reasons, TTR (0.5–1.0 IU/mL) was not reached in 32 neonatal VTE episodes. In seven neonates, no dosage adjustments and, therefore, no anti-Xa level measurements were performed as partial or complete thrombus resolution was observed with subtherapeutic levels of nadroparin. In three neonates, an anti-Xa level of 0.4 was accepted as TTR could not be reached despite many dosage adjustments. Nadroparin treatment was stopped in several neonates before reaching TTR due to subcutaneous catheter complications (*n* = 5), switch to UFH or recombinant tissue plasminogen activator (rTPA) (*n* = 4), other diagnosis (*n* = 2), minor bleeding (*n* = 1), death (*n* = 1) and allergic skin reaction (*n* = 1). In one neonate, nadroparin therapy was switched to prophylactic dosage because of thrombocytopenia. Five neonates were discharged to another hospital before reaching TTR. In one neonate, TTR was not reached within 21 days after start nadroparin therapy. Anti-Xa levels were not measured in one neonate after dosage adjustment.

### 3.3. Effectiveness Endpoints

During treatment of nadroparin, two neonates died as a result of their underlying disease. No therapy-related deaths were observed during the treatment period.

Data about recurrent thrombosis after start of nadroparin therapy were available in 61 neonatal VTEs, as in two neonates diagnosis of cerebral sinovenous thrombosis was withdrawn and one neonate was transferred to another hospital before follow-up ultrasonography was performed.

In 6 neonates (9.8%), recurrent thrombosis occurred during nadroparin therapy: thrombus extension in four and a new symptomatic VTE in two neonates. Patient characteristics are shown in Table 3. Clinical symptoms of recurrent thrombosis were only observed in two neonates with new VTE (patients 5 and 6 in Table 3).

Data about clot resolution at the time or shortly after the stop of nadroparin therapy were available in 41 neonatal VTEs. Follow-up ultrasonography was missing due to various reasons such as transfer to other hospitals and death. In addition, some neonatal VTEs were excluded for this outcome parameter while prior to nadroparin therapy, rTPA and UFH was given or nadroparin therapy was switched to UFH or dalteparin. Complete clot resolution was observed in 31 of 41 (75.6%) neonatal VTEs. TTR was reached in 18 of 31 (58.1%) neonatal VTEs in which complete clot resolution was observed.

### 3.4. Safety Endpoints

During administration of nadroparin treatment, none of the 61 neonates developed major bleeding. In three ELBW neonates, CRNMBs were observed. These CRNMBs were caused by the administration of nadroparin through subcutaneous catheters. The neonates suffered from large hematomas around the subcutaneous catheter, which resulted in red blood cell transfusion or vitamin K supplementation to restore homeostasis in two of them. In the third neonate, a surgical puncture for decompression of the hematoma was performed. Minor bleeding during treatment of nadroparin was found in 10 VTEs. Six of these minor bleedings were related to the use of subcutaneous catheters.

Two neonates developed an allergic skin reaction after administration of nadroparin. No anaphylactic reactions were observed during therapy.

Subcutaneous catheter complications occurred in 10 neonatal VTE episodes. In six ELBW and three VLBW neonates, hematomas (CRNMB *n* = 3, minor bleeding *n* = 6) developed around the subcutaneous catheter injection place, and in one term neonate, cellulitis around the injection place was reported. Nadroparin therapy needed to be withdrawn in seven neonates to prevent further complications.

## 4. Discussion

This study demonstrates that only 50% of preterm and term neonates treated with nadroparin for VTE reached TTR. High dosages of nadroparin were needed to obtain anti-Xa levels between 0.5 and 1.0 IU/mL, which seemed to be safe as no major (intracranial) bleedings occurred. With a higher nadroparin starting dosage, more neonates succeeded in reaching TTR. Remarkably, complete clot resolution occurred in more than 40% of neonates, who did not reach TTR. Recurrent VTE, however, was observed in six neonates, of whom five had anti-Xa levels less than 0.5 IU/mL. Finally, 10 neonates developed CRNMBs, minor bleedings, or cellulitis due to the use of subcutaneous catheters.

These results are comparable to the prospective study by van Ommen et al., in which two preterm and 10 term neonates were treated with nadroparin therapy [12]. Six of them (50%) reached TTR with a mean dosage of 224 ± 21 IU/kg/12 h. An explanation for high daily dosages of nadroparin in neonates is given by Laporte et al. [17]. They showed that characteristics of pediatric patients may influence the pharmacokinetic parameters of nadroparin since clearance and volume of distribution depend on weight and age. Neonates have a higher clearance of nadroparin and a larger volume of distribution than older children. Since nadroparin is soluble in water, neonates need higher dosages of nadroparin to reach TTR than older children. In addition, as a result of developmental hemostasis neonatal antithrombin levels are lower than in adults. For other LMWHs, higher dosages/kg are required in neonates, as well [11]. Although TTR was reached more often in group 2 with higher starting dose, median time to reach TTR was not significantly different between the groups. This might be explained by delay of venous blood sampling for anti-Xa levels after nadroparin dose adjustment.

In our study, TTR was reached in ELBW and VLBW neonates significantly less often directly after initial nadroparin dose, while median nadroparin dosages to reach TTR did not differ significantly between both groups. Physicians might have been more cautious in ELBW and VLWB neonates to increase nadroparin dosages due to increased bleeding risk. In addition, blood sampling for anti-Xa level measurements in ELBW and VLBW neonates are difficult to execute, resulting in less frequent anti-Xa level measurements and dosage adjustments to reach TTR. Finally, due to subcutaneous catheter bleedings, nadroparin therapy was stopped in five ELBW neonates before reaching TTR.

This study showed a relatively high frequency of recurrent VTE (9.8%) compared to an overall frequency of recurrent VTE of 3.2% in children 0–18 years old [11]. All recurrent VTE were observed in the first two weeks of treatment. In two neonates, VTE extension was diagnosed after five days of treatment. It might be possible that small thrombus extension is a common finding in the first days after diagnosis of neonatal VTE despite start of treatment. Five out of six neonates with recurrent VTE had anti-Xa levels less than 0.5 IU/mL, which might reflect the importance of reaching TTR as soon as possible. On the other hand, clot resolution occurred in more than 40% of the neonates who did not reach TTR at all. The currently used TTR in neonates and children is extrapolated from adult studies and recommendations. Whether it is also applicable to neonates, is not well known [5,18,19,20]. Some studies suggest that, especially in neonates and infants, LMWH administration could be effective without achieving the current TTR due to decreased thrombin generation compared to adults [12,21,22,23,24,25,26,27]. Our study partly confirms this hypothesis, as clot resolution occurred in 75% of neonatal VTEs, while TTR was not reached in more than 40% of these cases. Nevertheless, five neonates with suboptimal anti-Xa levels developed recurrent VTE. So radiological follow-up during anti-thrombotic therapy is extremely important in neonatal VTE.

This current study showed that high therapeutic nadroparin dosages in preterm and term neonates seem to be safe, if nadroparin is not administered through a subcutaneous catheter. None of the 61 neonates developed major (intracranial) bleeding while receiving nadroparin therapy. Malowany et al. reviewed all studies between 1980 and 2007 in which enoxaparin was used to treat neonates. The overall major bleeding rate was 4% (9 of 217 neonates) [21]. In later studies, reported bleeding rates ranged from 0% to 4% in pediatric patients [11,25,28,29]. Unfortunately, in nine ELBW and VLBW neonates, hematomas developed around the subcutaneous catheter injection place. One term neonate developed cellulitis. The occurrence of severe hemorrhagic and other subcutaneous catheter complications in ELBW and VLBW neonates treated with LMWH has been reported in previous studies [6,21,30,31,32,33]. Therefore, the use of these catheters should be discouraged, especially in these small neonates.

This study has several limitations, including the retrospective design of the study. As a result, it was not always known if blood samples were drawn exactly 4 h post-dosage. This has probably influenced the monitored anti-Xa levels. Furthermore, anti-Xa level measurements were not always performed after two administrations of nadroparin. Especially in ELBW neonates, anti-Xa levels were not regularly checked after dosage adjustment of nadroparin. In addition, some patients were discharged to another hospital before TTR was reached. As a consequence, some data about clot resolution was missing, and a few patients were lost to follow-up. Furthermore, it is unknown how many of the 61 neonates used a subcutaneous catheter. In group 1, before the protocol switch in 2014, pediatric hematologists already started with higher initial nadroparin dosages than advised in the protocol. Still, median initial dosage was significantly higher in group 2. The protocol switch may have incited pediatric hematologists to better try to reach TTR in neonates with VTE. This may have contributed to the increased number of neonates reaching TTR in group 2.

## 5. Conclusions

In conclusion, preterm and term neonates require a relatively high nadroparin dosage to reach TTR without major (intracranial) bleeding complications. In only half of the neonatal VTEs, TTR was reached. Nevertheless, clot resolution still occurred in about 40% of neonatal VTEs in which TTR was not reached. However, the frequency of recurrent VTE was relatively high. Therefore, nadroparin therapy should be personalized in neonates. Prospective studies are needed to investigate which neonates are at risk for recurrent thrombosis and need to reach TTR as soon as possible. The use of subcutaneous catheters should be discouraged, especially in ELBW and VLBW neonates.

## Figures and Tables

**Table 1:** Baseline characteristics of 64 VTE (venous thromboembolism) episodes in 61 preterm and term neonates treated with nadroparin.

Characteristics	Total Group	Group 1	Group 2	*p*-Value
Sex				
Male	*n* = 34 (55.7%)	*n* = 15 (55.6%)	*n* = 19 (55.9%)	0.98 ^a^
Female	*n* = 27 (44.3%)	*n* = 12 (44.4%)	*n* = 15 (44.1%)	
Gestational Age				
Median, range weeks	30.3 (24–40.7)	26.6 (24–40.7)	32,7 (24.4–39.7)	0.067 ^b^
<28 weeks	*n* = 25 (41.0%)	*n* = 15 (55.6%)	*n* = 10 (29.4%)	
28–32 weeks	*n* = 10 (16.4%)	*n* = 3 (11.1%)	*n* = 7 (20.6%)	
32–37 weeks	*n* = 9 (14.8%)	*n* = 4 (14.8%)	*n* = 5 (14.7 %)	
≥37 weeks	*n* = 17 (27.9%)	*n* = 5 (18.5%)	*n* = 12 (35.3%)	
Birth Weight				
Median, range in kilogram	1.16 (0.4–4.35) ^c^	0.96 (0.40–4.35) ^c^	1.60 (0.43–3.90)	0.027 ^b^
Weight at start Nadroparin				
Median, range in kilogram	1.80 (0.53–4.30)	1.09 (0.53–4.30)	2.45 (0.72–3.89)	0.007 ^b^
Age at start Nadroparin				
Median, range in days	16 (1–124)	14 (1–58)	16 (3–124)	0.352 ^b^
Number of VTE episodes	*n* = 64 ^d^	*n* = 28	*n* = 36	
Location thrombus				
Right atrium	*n* = 22 (34.4%)	*n* = 14 (50.0%)	*n* = 8 (22.2%)	
Cerebral sinovenous thrombosis	*n* = 12 (18.8%) ^e^	*n* = 8 (28.6%)	*n* = 4 (11.1%) ^e^	
Subclavian/Jugular vein	*n* = 8 (12.5%)	*n* = 1 (3.6%)	*n* = 7 (19.4%)	
Renal vein	*n* = 6 (9.4%)	*n* = 2 (7.1%)	*n* = 4 (11.1%)	
Deep venous thrombosis of the legs	*n* = 6 (9.4%)	*n* = 1 (3.6%)	*n* = 5 (13.9%)	
Inferior caval/Umbilical/Hepatic vein	*n* = 5 (7.8%)	*n* = 0 (0%)	*n* = 5 (13.9%)	
Deep venous thrombosis of the arms	*n* = 2 (3.1%)	*n* = 0 (0%)	*n* = 2 (5.6%)	
Portal vein	*n* = 2 (3.1%)	*n* = 1 (3.6%)	*n* = 1 (2.8%)	
Pulmonary embolism	*n* = 1 (1.6%)	*n* = 1 (3.6%)	*n* = 0 (0%)	
Associated risk factors				
Central venous catheters	*n* = 47 (73.4%)	*n* = 16 (57.1%)	*n* = 31 (86.1%)	0.036 ^a^
(Suspicion of) Sepsis	*n* = 24 (37.5%)	*n* = 11 (39.3%)	*n* = 13 (36.1%)	0.801 ^a^
Congenital heart disease	*n* = 13 (20.3%)	*n* = 2 (7.1%)	*n* = 11 (30.6%)	0.028 ^a^
Initial dose of Nadroparin	146.6 (60.9–228.6)	117.9 (60.9–167.0)	166.1 (121.4–228.6)	0.000 ^b^
Median, range (IU/kg/12 h)				

^a^ Chi-square test; ^b^ Mann Whitney *U* test; ^c^ In one neonate birth weight is missing; ^d^ Three neonates were enrolled twice for the pharmacokinetic part of the study; ^e^ In two neonates diagnosis of cerebral sinovenous thrombosis was withdrawn. IU, International Unit.

**Table 2:** Therapeutic target range.

	Total Group	Group 1	Group 2	*p*-Value
	64 VTE episodes	28 VTE episodes	36 VTE episodes	
Initial dose of Nadroparin				
Median, range (IU/kg/12 h)	146.6 (60.9–228.6)	117.9 (60.9–167.0)	166.1 (121.4–228.6)	0.000 ^a^
Reaching TTR ^b^ after initial dose				
number	11 (17.2%)	*n* = 6 (21.4%)	*n* = 5 (13.9%)	0.513 ^c^
TTR achievement, number				
All weight categories	32 (50.0%)	*n* = 10 (35.7%)	*n* = 22 (61.1%)	0.077 ^c^
Weight > 1.5 kg at start Nadroparin	22 (34.4%)	*n* = 6 (21.4%)	*n* = 16 (44.4%)	
Weight < 1.5 kg at start Nadroparin	5 (7.8%)	*n* = 2 (7.1%)	*n* = 3 (8.3%)	
Weight < 1.0 kg at start Nadroparin	5 (7.8%)	*n* = 2 (7.1%)	*n* = 3 (8.3%)	
Time to reach TTR ^d^				
Median, range in days	3.5 (1–21)	3 (1–21)	4 (1–15)	0.734 ^a^
Dose adjustments to reach TTR				
Median, range in number	1 (0–4)	0 (0–2)	2 (0–4)	0.025 ^a^
Dose to reach TTR				
Median, range (IU/kg/12 h)	197.0 (97.9–330.3)	132.8 (97.9–197.4)	206.2 (149.2–330.3)	0.000 ^a^

^a^ Mann Whitney *U* test; ^b^ Therapeutic Target Range; ^c^ Chi-square test; ^d^ TTR should have been reached in 21 days after start of nadroparin therapy.

**Table 3:** Characteristics of 6 neonates diagnosed with recurrent thrombosis during nadroparin therapy.

Characteristics	Patient 1	Patient 2	Patient 3 ^a^	Patient 4	Patient 5	Patient 6 ^a^
Group 1 (2007–2013) or 2 (2014–2017)	1	1	1	2	2	2
Gestational age (weeks ^days^)	24^+3^	30^+3^	33^+4^	39^+5^	26^+1^	37^+5^
Weight at time of diagnosis rVTE ^b^ (kilogram)	1.00	2.16	1.80	3.68	0.83	3.07
Age at start nadroparin (days)	32	58	17	28	11	9
Duration nadroparin therapy before diagnosis rVTE ^b^ (days)	11	12	3	5	4	2
Location thrombus	Right atrium	Right atrium	Right atrium	CSVT ^c^	Right atrium	Right jugular vein
rVTE ^b^ Extension—New						
	Extension	Extension	Extension	Extension	New ^d^	New ^e^
Associated Risk Factors						
Central venous catheter	+	+	+	+	+	+
Infection	-	-	+ ^f^	-	+ ^g^	-
Congenital heart disease	-	-	-	-	-	+
Initial dose of nadroparin (IU/kg/day)	220	234	300	264	373	293
Dose of nadroparin at diagnosis rVTE ^b^ (IU/kg/day)	230	336	300	353	474	391
Anti-Xa level at diagnosis rVTE^b^ (IU/mL)	- ^h^	- ^i^	<0.10	0.23	0.77	0.20
Changes in therapy after diagnosis rVTE	rTPA ^j^ followed by nadroparin ↑ ^k^	Nadroparin ↑ ^k^	UFH ^l^	Nadroparin ↑ ^k^	rTPA ^j^ followed by nadroparin ↑ ^k^	UFH ^l^

^a^ This patient was included twice in the study; ^b^ Recurrent venous thrombotic episode; ^c^ Cerebral sinovenous thrombosis; ^d^ Pulmonary embolism; ^e^ Left jugular vein; ^f^ *Staphylococcus aureus* sepsis and endocarditis; ^g^ Coagulase Negative *Staphylococcus* sepsis; ^h^ Anti-Xa level was 0.46 IU/mL one week before diagnosis of rVTE; ^i^ Anti-Xa level was 0.28 IU/mL five days prior to diagnosis of rVTE; ^j^ Recombinant tissue plasminogen activator; ^k^ Dosage increase; ^l^ Unfractionated heparin.

## Data Availability

The data presented in this study are available on request from the corresponding author. The data are not publicly available due to privacy reasons.

## Abbreviations

CRNMB	clinically relevant non-major bleeding
ELBW	extremely low birth weight
LMWH	low-molecular-weight-heparin
TTR	therapeutic target range
UFH	unfractionated heparin
VTE	venous thromboembolic event
VLBW	very low birth weight
